# Candidemia in a General Hospital in Kuwait: Epidemiology, Species Distribution, Risk Factors, and Antifungal Susceptibility Patterns over a 10-Year Period (2015–2024)

**DOI:** 10.3390/jof11090670

**Published:** 2025-09-12

**Authors:** Khalifa Al Benwan, Sarah Ahmed, Dalal Al Banwan, Maria John

**Affiliations:** 1 Department of Microbiology, College of Medicine, Kuwait University, P.O. Box 24923, Safat, Kuwait City 13110, Kuwait; sarah.ahmed@ku.edu.kw; 2 Department of Microbiology, Al-Amiri Hospital, P.O. Box 4077, Safat, Kuwait City 13041, Kuwait; drmariajohn@gmail.com; 3 Department of Statistics and Operations Research, College of Science, Kuwait University, P.O. Box 5969, Safat, Kuwait City 13060, Kuwait; dalal.albanwan@ku.edu.kw

**Keywords:** candidemia, *Candida* species, epidemiology, risk factors

## Abstract

This 10-year retrospective observational study (2015–2024) conducted at Al-Amiri Hospital in Kuwait aimed to analyze the epidemiology, species distribution, and key risk factors associated with *Candida* bloodstream infections. Data were collected on patient demographics and clinical risk factors, and the distribution of *Candida* species was determined based on isolates recovered from patients with confirmed candidemia. Multivariate logistic regression was performed to identify factors associated with candidemia outcomes. Cases significantly increased from 33 (2015–2016) to 93 (2023–2024), predominantly affecting elderly patients (≥65 years) and intensive care unit (ICU) admissions. A shift in species distribution was observed, with a decline in *Candida albicans* and a marked increase in *Candidozyma auris* (formerly *Candida auris*) and *C. parapsilosis*. Antifungal susceptibility patterns were species-specific: *C. albicans*, *C. parapsilosis*, and *C. tropicalis* remained highly susceptible to all tested antifungals, while *Nakaseomyces glabratus* (formerly *Candida glabrata)* showed fluconazole resistance in 25% of isolates. *C. auris* exhibited resistance to fluconazole (97%) and variable resistance to echinocandins and voriconazole. Echinocandins retained broad-spectrum activity across most species. Independent risk factors included ICU admission, advanced age, and comorbidities. *N. glabratus* and *C. auris* infections were linked to higher mortality. This study highlights the growing candidemia burden in Kuwait, driven by emerging non-*albicans Candida* (NAC) spp. and related species. Early species identification and susceptibility testing are crucial for effective treatment and improved outcomes, necessitating enhanced infection control and antifungal stewardship.

## 1. Introduction

Candidemia, a bloodstream infection caused by *Candida* spp. and related species, is a major concern in healthcare settings worldwide. It is associated with high morbidity and mortality rates, particularly among critically ill and immunocompromised patients [1,2]. The global epidemiology of candidemia has changed significantly in recent years, with a notable increase in incidence and a shift from *Candida albicans* to non-*albicans Candida* (NAC) spp. and related species [3]. This shift is largely attributed to the widespread use of antifungal agents, increased hospitalization rates, and the growing prevalence of invasive medical procedures [4]. Globally, the estimated annual incidence of candidemia exceeds 500,000 cases, with approximately 250,000 associated deaths [5]. Notably, substantial variability in incidence has been observed across countries and among different populations at risk for invasive candidiasis.

In Kuwait, candidemia has emerged as a major nosocomial infection, with reported incidence rates ranging from approximately 2 to 5 cases per 100,000 population [6]. However, higher estimates of 6.8 per 100,000 have been suggested, necessitating robust surveillance strategies in the country [7]. A comprehensive 12-year study on candidiasis in Kuwait identified *C. albicans* and *Candida parapsilosis* as the most frequently isolated species, accounting for 37% and 34% of cases, respectively [8]. Supporting this observation, a nationwide survey on candidemia in Kuwait reported a similar distribution pattern. However, the survey also highlighted a concerning finding: an increasing incidence of multidrug-resistant *Candidozyma auris*, with 33 confirmed cases [6]. *C. auris* poses a serious threat to public health due to its resistance to the commonly used antifungal agents, its persistence in hospital environments, and its potential to cause outbreaks that are difficult to control [9].

The emergence of *C. auris* underscores the critical need for continuous surveillance of *Candida* spp. and related species distribution, particularly within major healthcare centers, where vulnerable patient populations are treated. For example, while the nationwide survey identified Al-Amiri Hospital as having a comparatively lower incidence of candidemia than other hospitals in Kuwait [6], this observation warrants in-depth investigation. Al-Amiri is a leading general hospital, with a capacity exceeding 400 beds, and it serves an estimated 400,000 patients annually across a broad range of medical and surgical specialties. The detection of *C. auris* in this setting, given its ability to persist on surfaces for extended periods, raises concerns about potential underreporting and the likelihood of increasing infection rates over time [9].

In light of these concerns, the present study aims to provide in-depth epidemiological insights into candidemia at Al-Amiri Hospital (AAH), a major hospital in Kuwait, over a ten-year period (2015–2024). Through detailed multivariate analyses, this study seeks to identify key risk factors associated with candidemia, including ICU admissions, comorbidities, and the use of invasive medical devices. Additionally, the antifungal susceptibility patterns of the isolated *Candida* spp. and related species were evaluated. By understanding these trends, healthcare providers can implement targeted interventions, strengthen infection control practices, and optimize antifungal therapy. Ultimately, this research seeks to reduce the burden of candidemia, improve patient survival, and inform public health strategies in hospital settings.

## 2. Materials and Methods

### 2.1. Study Design and Setting

This retrospective observational study was conducted in the Department of Microbiology at AAH, Kuwait, a 400-bed teaching hospital with a 15-bed surgical intensive care unit, a 25-bed medical intensive care unit, and specialized urology, renal dialysis, and kidney transplant units. The hospital serves approximately 400,000 people of various nationalities and provides clinical laboratory services to 22 polyclinics. This study included all episodes of candidemia recorded between 1 January 2015 and 31 December 2024.

### 2.2. Case Identification and Classification of Candidemia Episodes

Patients’ demographic data and the location of care at the onset of candidemia (or fungemia for other yeasts) were recorded. Blood samples were collected from each patient after obtaining verbal consent in accordance with hospital protocols and ethical guidelines, as part of routine patient care and diagnostic work-up. A candidemia episode was defined as the first positive blood culture yielding a specific *Candida* spp. or related species. Any additional positive cultures for the same species obtained within 30 days were considered part of the same episode to avoid duplication. A recurrent episode was defined as a new *Candida*-positive blood culture—caused by the same or a different species—occurring more than 30 days after the initial episode, provided there was documented clinical resolution of the prior infection and at least one intervening negative blood culture confirming clearance. Mixed candidemia cases were defined as those involving more than one *Candida* species during the same episode. Due to the retrospective design and limitations in documentation, detailed information regarding the number of blood culture sets, their timing relative to clinical symptoms (e.g., fever or chills), and the anatomical site of collection was not uniformly available and was therefore not included in the analysis.

### 2.3. Isolation, Species-Specific Identification, and Antifungal Susceptibility Testing of Yeast Isolates

The microbiology laboratory at AAH utilizes automated blood culture systems, including BACTEC 9240 (Becton Dickinson, Sparks, MD, USA), BacT/Alert 3D (bioMérieux, Marcyl’-Étoile, France), and VersaTREK™ 240 (Thermo Fisher Scientific, Waltham, MA, USA), for the isolation of yeasts from blood specimens. Species-level identification was initially performed using phenotypic methods with the VITEK 2 yeast identification system (bioMérieux, Marcy-l’Étoile, France) for assimilation profiling. Definitive identification was then confirmed using MALDI-TOF MS (VITEK^®^ MS; bioMérieux, Marcy-l’Étoile, France) for protein profiling, following the manufacturer’s instructions, and in selected cases, molecular assays. Briefly, fresh yeast colonies were applied to a target plate, overlaid with a matrix solution containing α-cyano-4hydroxycinnamic acid (CHCA), air-dried, and analyzed using the VITEK^®^ MS system. Identification was achieved by comparing the generated mass spectra against the IVD (in vitro diagnostic) reference database, enabling species-level classification based on unique protein fingerprints [10,11].

All growth-positive blood cultures were forwarded to the Mycology Reference Laboratory (MRL), where isolates were subcultured on Sabouraud dextrose agar for phenotypic identification based on colony characteristics, as previously described [12]. In accordance with laboratory protocols, all *Candida* bloodstream isolates were preserved at –80 °C in a strain archive and selectively subjected to molecular testing for epidemiological or research purposes, particularly in cases of suspected *C. auris*, rare or ambiguous species, or isolates exhibiting unusual antifungal resistance profiles.

For this retrospective study, molecular methods were applied to selected archived isolates to confirm identification and support epidemiological analysis. A multiplex PCR assay was used to differentiate *C. parapsilosis* sensu stricto from *C. orthopsilosis* and *C. metapsilosis*, as previously described [13]. PCR amplification of rDNA with species-specific primers was performed to confirm all *C. auris* and *Clavispora lusitaniae* (formerly *Candida lusitaniae*) isolates [12,14]. Additionally, isolates with atypical phenotypic features or antifungal resistance underwent sequencing of the internal transcribed spacer (ITS) region of rDNA using panfungal primers [15], providing definitive molecular identification.

Antifungal susceptibility testing (AFST) was conducted on a representative subset of 100 out of 323 *Candida* bloodstream isolates using the Sensititre^®^ YeastOne™ system (Thermo Fisher Scientific, USA), which provides quantitative minimum inhibitory concentration (MIC) values in pre-dosed, colorimetric 96-well plates. Selection was based on resource limitations and aimed to ensure proportional species representation, temporal trends, and clinical relevance, including isolates from high-risk settings and treatment failures. The antifungal agents tested included fluconazole, itraconazole, posaconazole, voriconazole, anidulafungin, caspofungin, micafungin, amphotericin B, and 5-flucytosine.

Following the manufacturer’s instructions, the fungal inoculum was prepared to achieve a final organism density of approximately 1.5–8 × 10^3^ CFU/mL (target range: 1–5 × 10^3^ CFU/mL). Plates were incubated at 35 °C for 24–48 h in ambient air (non-CO_2_). MICs were determined visually after 24–48 h of incubation, defined as the lowest antifungal concentration that inhibited growth, indicated by the absence of a color change from blue to red or purple, in accordance with the manufacturer’s guidelines [16]. Interpretation of MICs for *C. albicans*, *N. glabratus*, *C. parapsilosis*, *C. tropicalis*, *Pichia kudriavzevi*, formerly known as *Candida krusei, C. dubliniensis*, and *C. lusitaniae* was based on species-specific Clinical and Laboratory Standards Institute (CLSI) breakpoints, following M27 and M44 guidelines [17]. Isolates were categorized as susceptible (S), susceptible-dose dependent (SDD), intermediate (I), or resistant (R), as applicable.

For *C. auris*, which lacks CLSI-defined breakpoints, tentative resistance thresholds derived from CLSI and EUCAST epidemiological cutoffs were used: fluconazole (≥32 µg/mL), voriconazole (≥2 µg/mL), posaconazole (≥2 µg/mL), itraconazole (≥2 µg/mL), echinocandins (≥4 µg/mL), amphotericin B (≥2 µg/mL), and 5-flucytosine (≥32 µg/mL) [18]. Quality control was performed using *P. kudriavzevii* ATCC 6258 and *C. parapsilosis* ATCC 22019, with results consistently falling within established CLSI quality control ranges (M27M44S) [17].

### 2.4. Statistical Analyses

Patients’ demographic data and clinical variables were analyzed using descriptive statistics. Categorical variables were expressed as frequencies and percentages, and continuous variables as medians with interquartile ranges (IQRs). Comparisons between groups were performed using Pearson’s Chi-square test or Fisher’s exact test, as appropriate. The clinical variables included in the analysis were gender, age, cancer, kidney disease, central venous access (CVA), ischemic heart disease (IHD), hypertension (HTN), diabetes mellitus, dyslipidemia, recent surgery, ventilator use, intravenous nutrition, central venous catheterization, and urinary catheterization. These variables were subsequently incorporated into the multivariate logistic regression model to identify species-specific risk factors for candidemia and predictors of mortality. Odds ratios (ORs) with 95% confidence intervals (CIs) and *p*-values were calculated, with *p* < 0.05 considered statistically significant.

## 3. Results

### 3.1. Demographic and Epidemiological Trends

As shown in Table 1, the incidence of candidemia at Al-Amiri Hospital significantly increased from 33 cases in 2015–2016 to 93 cases in 2023–2024 (χ^2^ = 22.14, *p* = 0.0002), representing a nearly threefold rise. Gender distribution was nearly equal throughout the study period (male: 49.8%, female: 50.2%), with no significant difference in incidence over time (χ^2^ = 0.002, *p* = 0.96).

The ≥65-year age group consistently bore the highest burden of candidemia, increasing from 19 cases in 2015–2016 to 57 cases in 2023–2024. The 50–64-year age group also showed a steady rise, while younger age groups remained relatively stable. Infants (≤1 year) were not affected, and the 1–19 year age group was minimally affected, with only two cases recorded in 2023–2024.

By hospital location, ICU cases accounted for 36–47% of all candidemia cases across the study period, while ward cases represented a higher overall proportion (59%). A significant association was found between hospital unit and candidemia incidence (χ^2^ = 6.145, *p* = 0.013). Among ward cases, 29% occurred in medical wards, 23% in surgical wards, and 7% in hemodialysis units.

### 3.2. Distribution of Candida spp. and Related Species

**Figure 1:**
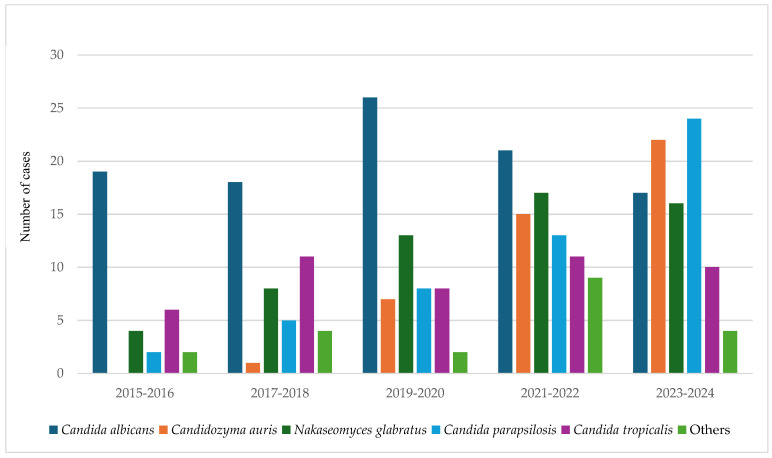
Temporal trends in the distribution of *Candida* spp. and related species causing candidemia at AAH, Kuwait (2015–2024). This bar chart shows the annual distribution of *Candida* spp. and related yeast species isolated from bloodstream infections over a 10-year period. The Y-axis represents the number of candidemia cases per species. *C. albicans* remained the most frequently isolated species, but its proportion declined markedly from 57.6% in 2015–2016 to 18.3% in 2023–2024. In contrast, non-*albicans* *Candida* (NAC) species showed a progressive increase, particularly *C. auris* (emerging from 0 to 22 cases) and *C. parapsilosis* (rising from 2 to 24 cases). Other species, including *N. glabratus* and *C. tropicalis*, were consistently detected, while rare isolates such as *P. kudriavzevii* and *K. ohmeri* appeared sporadically. Overall, the species distribution shift was statistically significant (χ^2^ = 32.85, *p* = 0.004), underscoring the rising burden of multidrug-resistant NAC spp. and related species and their clinical importance in candidemia.

As shown in Table 2, *C. albicans* remained the most frequently isolated species over the 10-year period, although its proportion gradually declined. In contrast, *C. auris* emerged as a notable pathogen, rising from 0 cases in 2015–2016 to 22 cases in 2023–2024. *N. glabratus*, *C. parapsilosis*, and *C. tropicalis* were consistently detected, with *C. parapsilosis* showing a marked increase (Figure 1). Rare species such as *P. kudriavzevii* and *Kodamaea ohmeri* were infrequently isolated. Candidemia was most prevalent in patients ≥ 65 years, followed by those aged 50–64 years, with fewer cases in younger adults (20–49 years). While *C. albicans* remained overall dominant, *N. glabratus* and *C. auris* were more frequently isolated in older patients in terms of absolute case numbers, although their relative proportions varied across age groups. This reflects the overall higher burden of candidemia in elderly populations and highlights the emergence of resistant NAC spp. and related species in this demographic.

Overall, *C. albicans* accounted for 101 cases, followed by *N. glabratus* (n = 58), *C. parapsilosis* (n = 52), *C. tropicalis* (n = 46), and *C. auris* (n = 45). Most infections occurred in patients aged ≥65 years (60%), with a slight predominance among females, while pediatric cases were uncommon.

### 3.3. Shifting Trends in Candida Species over Time

As shown in Figure 1 and Table 3, a significant shift occurred in the distribution of *Candida* species, with a decline in *C. albicans* and a rise in NAC spp. and related species over time (χ^2^ = 32.85, *p* = 0.004). *C. auris* showed the most dramatic increase, from 0 cases in 2015–2016 to 22 cases in 2023–2024. *C. parapsilosis* also rose significantly, from 2 cases to 24 over the same period. In contrast, *C. albicans* declined modestly from 19 to 17 cases.

**Figure 2:**
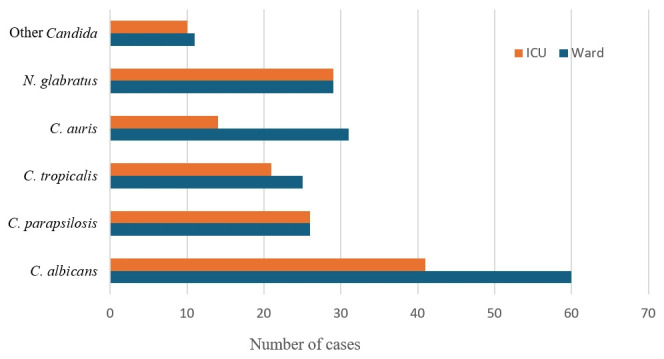
Distribution of *Candida* spp. and related species in wards and intensive care units (ICUs) at AAH, Kuwait (2015–2024). The chart shows the number of isolates for each *Candida* spp. and related species, highlighting *C. albicans* as the most common in both settings. Notably, *C. auris* was more prevalent among general ward patients, while *N. glabratus* and *C. parapsilosis* were evenly distributed at AAH from 2015 to 2024.

As presented in Figure 2, *C. albicans* remained the most common species isolated in both ICU and ward settings. However, *C. auris* was more prevalent among ward patients (31 cases) than ICU patients (14 cases). *N. glabratus* and *C. parapsilosis* were evenly distributed between the two locations, while *C. tropicalis* had a slightly higher prevalence in ward patients.

### 3.4. Antifungal Susceptibility Patterns of Candida Bloodstream Isolates

As shown in Table 4, antifungal susceptibility testing of *Candida* bloodstream isolates revealed some species-specific patterns. With the exception of *C. auris*, voriconazole, amphotericin B, 5-flucytosine, and echinocandins were effective against all identified species. Fluconazole resistance was observed in 14.3% of *C. albicans*, 25% of *N. glabratus*, and 8.3% of *C. parapsilosis*, while no resistance was detected in *C. tropicalis*. In contrast, *C. auris* exhibited near-universal resistance to fluconazole (97.4%) and moderate resistance to voriconazole (12.8%), based on CLSI breakpoint values. Resistance to echinocandins was also observed, ranging from 5.1% to 7.7% Additionally, low-level resistance to amphotericin B and 5-flucytosine (2.6% each) was observed in *C. auris* [19]. The geometric mean MIC values for echinocandins were consistently low across species, reinforcing their efficacy and supporting their continued use as first-line agents for candidemia.

### 3.5. Multivariate Analysis of Risk Factors for Species-Specific Candidemia and Mortality

Multivariate analysis identified several significant risk factors for species-specific candidemia and associated mortality (Table 5). For *C. albicans*, significant associations were found with cancer (OR: 5.94; *p* < 0.001), kidney disease (OR: 3.97; *p* = 0.001), ischemic heart disease (IHD) (OR: 4.43; *p* < 0.001), hypertension (HTN) (OR: 2.79; *p* = 0.005), diabetes mellitus (OR: 2.38; *p* = 0.012), dyslipidemia (OR: 2.79; *p* = 0.010), ventilator use (OR: 3.36; *p* = 0.016), and central venous catheterization (OR: 2.99; *p* = 0.048).

*C. auris* was negatively associated with cancer (OR: 0.33; *p* = 0.016), kidney disease (OR: 0.09; *p* < 0.001), HTN (OR: 0.31; *p* < 0.001), diabetes (OR: 0.44; *p* = 0.014), and dyslipidemia (OR: 0.29; *p* < 0.001), suggesting a distinct risk profile. It was also associated with reduced odds in urinary catheterized patients (OR: 0; *p* = 0.034).

For *C. parapsilosis*, central venous catheter use (OR: 4.18, 95% CI: 2.34–7.49, *p* < 0.001), surgery (OR: 2.91, 95% CI: 1.63–5.19, *p* = 0.008), total parenteral nutrition (OR: 3.71, 95% CI: 1.93–7.14, *p* = 0.002), and prolonged intravenous therapy over seven days (OR 2.43; 95% CI 1.36–4.34; *p* = 0.015) were the most significant risk factors, supporting the species’ well-established association with catheter-related and healthcare-acquired infections.

In terms of mortality, significant predictors included male gender (protective; OR: 0.60; *p* = 0.025), cancer (OR: 0.23; *p* < 0.001), kidney disease (OR: 2.62; *p* < 0.001), IHD (OR: 3.03; *p* < 0.001), HTN (OR: 1.71; *p* = 0.024), dyslipidemia (OR: 1.58; *p* = 0.043), ventilator use (OR: 1.91; *p* = 0.015), and absence of surgery (surgery protective; OR: 0.41; *p* < 0.001).

The median age of patients ranged from 58 years in *C. auris* cases to 75 years in *C. albicans*, with overall mortality occurring at a median age of 68 years. Male gender was not significantly associated with any specific *Candida* spp. and related species but was independently linked to lower overall mortality (OR: 0.60; *p* = 0.025). Mortality was significantly associated with underlying kidney disease, ischemic heart disease (IHD), hypertension, mechanical ventilation, and infection with NAC species (OR: 2.04; *p* = 0.011). In contrast, infection with *C. albicans* (OR: 0.20; *p* < 0.001) and the presence of cancer were associated with reduced mortality.

## 4. Discussion

This study represents the largest and most detailed single-center analysis of candidemia in Kuwait to date, documenting a nearly threefold increase in cases at AAH over the past decade. These findings underscore the growing burden of invasive fungal infections and align with global epidemiological trends [20,21]. The statistically significant rise in cases suggests a combination of evolving hospital practices, increased patient vulnerability, and shifts in *Candida* epidemiology [21].

The nearly equal male-to-female ratio observed in our study is consistent with findings from both global and regional investigations [6,22]. Although some reports have documented a slight male predominance [22], recent studies from Gulf countries increasingly support a balanced 1:1 gender distribution in candidemia cases [23]. While subtle sex-based immunological differences may affect susceptibility in specific populations, the overall risk appears comparable between men and women when adjusted for underlying comorbidities [24]. This understanding has important implications for both clinical practice and epidemiological surveillance. It emphasizes that prevention strategies and diagnostic vigilance for candidemia should be implemented uniformly across all patients, regardless of gender [25].

Our findings reaffirm that older adults (≥65 years) are at highest risk for candidemia, consistent with global studies [26]. This increased susceptibility in the elderly is attributed to factors such as age-related immune dysfunction, multiple comorbidities, frequent hospitalizations, and prolonged use of invasive medical devices. For instance, a study analyzing 175 episodes of candidemia in elderly patients reported a mean age of 76.4 years, with cancer and diabetes mellitus being the most common underlying conditions [26]. Additionally, the rising incidence among middle-aged adults (50–64 years) may be linked to chronic illnesses and invasive treatments, highlighting a broader shift in candidemia epidemiology toward both elderly and other high-risk populations [25,26]. These trends necessitate targeted surveillance and prophylactic strategies for both age groups.

Interestingly, general ward patients constituted a higher proportion of candidemia cases compared to ICU patients, challenging the traditional association of candidemia with intensive care settings. This shift may reflect better ICU-based infection control and an expanding candidemia burden among non-critically ill, comorbid patients [27,28]. Recent studies in Italy and Korea similarly report increased candidemia rates in general wards, indicating a broader hospital-wide distribution [29,30].

Species distribution trends in our study align with both regional and international reports documenting a significant epidemiological shift from *C. albicans* to NAC spp. and related species, notably *C. auris*, *C. parapsilosis*, and *N. glabratus* [31,32]. Over the past decade, Gulf hospitals—including those in Kuwait, Saudi Arabia, and Bahrain—have reported a rising prevalence of NAC spp. and related species as leading causes of hospital-associated candidemia. In Kuwait, a 2018 national survey found that NAC spp. and related species comprised the majority of bloodstream isolates, with *C. parapsilosis*, *C. tropicalis*, *C. auris*, and *N. glabratus* collectively dominating the species landscape [6]. Notably, *C. auris* emerged rapidly, nearly matching *N. glabratus* in frequency, a trend echoed across neighboring Gulf countries [22,23].

In our study, *C. auris* was more commonly isolated from ward patients than ICU patients, suggesting hospital-wide transmission dynamics rather than ICU-specific clustering. As highlighted by Arendrup and Patterson [33], effective containment of *C. auris* requires rigorous environmental cleaning and enhanced diagnostic capabilities. On the other hand, the increasing prevalence of *C. parapsilosis* in our setting may reflect a rise in catheter-associated bloodstream infections, given its strong affinity for biofilm formation on medical devices [34]. This reinforces the importance of meticulous catheter care, especially among patients receiving total parenteral nutrition or prolonged intravenous therapy. Additionally, *N. glabratus* was frequently associated with prior fluconazole exposure and underlying malignancies [11], underscoring the role of antifungal selection pressure and the need for targeted surveillance and stewardship interventions.

Antifungal susceptibility testing of bloodstream *Candida* isolates from our study provided critical insights into resistance patterns across species. The findings reveal notable variability, particularly among NAC spp. and related species, with implications for empirical therapy and antifungal stewardship in Kuwait [21]. *C. albicans* demonstrated generally favorable susceptibility patterns, with fluconazole resistance observed in only 14.3% of isolates, while all isolates remain fully susceptibility to amphotericin B and echinocandins [32,35]. In contrast, *C. auris* exhibited a markedly concerning resistance profile—fluconazole resistance was nearly universal (97.4%), with additional resistance to voriconazole (12.8%) and echinocandins (5.1–7.7%). These findings are consistent with previous reports from Kuwait and neighboring countries [32] and reinforce the current recommendation of echinocandins as the first-line treatment for *C. auris* candidemia [36,37].

*N. glabratus* showed moderate fluconazole resistance (25%). While echinocandin resistance was not observed in our study, ongoing surveillance remains important given the global emergence of *N. glabratus* strains with FKS mutations [21]. These findings underscore the importance of local antifungal susceptibility data to guide therapy [38]. Both *C. parapsilosis* and *C. tropicalis* displayed excellent susceptibility to all antifungal agents tested. This susceptibility pattern supports the continued efficacy of azoles and echinocandins for these species [12]. Overall, our findings reinforce the importance of species-level identification and local susceptibility data [37,39],.

The comparative multivariate analysis highlights notable differences in risk factors associated with candidemia caused by *C. albicans, N. glabratus*, and *C. auris*, as well as predictors of mortality. Male gender was not significantly associated with infection by any of the three major *Candida* species; however, it was significantly associated with a lower risk of mortality (OR = 0.60, *p* = 0.025), suggesting a possible survival advantage [40]. Cancer was strongly associated with increased risk of *C. albicans* and *N. glabratus*, consistent with previous studies reporting that malignancy, chemotherapy, and central line use predispose patients to these infections [38]. Interestingly, cancer was inversely associated with *C. auris* and mortality, which may be due to differences in care intensity or early identification in oncology settings [41].

Hypertension was associated with both *C. albicans* and *N. glabratus*, and with increased mortality, but showed no significant association with *C. auris*. Furthermore, diabetes mellitus and dyslipidemia increased the odds of *C. albicans* and *N. glabratus* candidemia, aligning with studies highlighting metabolic disorders as risk factors for invasive candidiasis. However, their inverse association with *C. auris* may reflect different transmission dynamics and environmental persistence rather than classical host susceptibility [37]. Surgical history was a strong predictor of *N. glabratus* infection (OR = 6.08), in line with prior evidence linking surgical patients with increased risk for *N. glabratus* fungemia due to gut translocation or mucosal barrier disruption. Paradoxically, surgery was inversely associated with mortality (OR = 0.41), potentially reflecting closer monitoring and more timely interventions in post-operative patients [37]. Mechanical ventilation was associated with both *C. albicans* and mortality, reinforcing its role as a surrogate for critical illness severity. Central venous catheterization showed a strong association with *N. glabratus* (OR = 10.57), a finding widely recognized in both local and international studies of catheter-related candidemia [39].

Importantly, *C. auris* displayed an inverse association with many traditional risk factors such as cancer, kidney disease, diabetes, and dyslipidemia, underscoring its distinct epidemiologic behavior. Its spread has been predominantly linked to nosocomial outbreaks, environmental contamination, and poor disinfection practices, as observed in multiple Gulf-based reports [35].

*C. parapsilosis* showed the strongest associations with intravascular devices, surgery, and parenteral nutrition, reinforcing its role as a healthcare-associated pathogen. Mortality was highest among patients with chronic kidney disease, ischemic heart disease, and those requiring mechanical ventilation, highlighting the impact of host comorbidities and critical illness on outcomes.

Although COVID-19 status was not specifically assessed in this study, the modest increase in candidemia cases during 2020–2022 coincided with the pandemic. However, this trend was already evident prior to the pandemic and persisted thereafter, suggesting that broader factors—such as ICU burden, increased use of invasive devices, and the emergence of drug-resistant species—were the primary drivers of candidemia in our setting.

This study has several limitations, including its retrospective design, which may introduce reporting bias and incomplete clinical data. This study lacked consistent data on the primary source of infection preceding candidemia, which limited the ability to analyze source-specific risk factors such as catheter-related or intra-abdominal infections. Antifungal susceptibility testing was limited to a representative subset of isolates due to resource constraints. COVID-19 status was not consistently available in the dataset, limiting the evaluation of its potential impact on candidemia trends. The single-center setting may also affect the generalizability of findings to other institutions. Additionally, advanced molecular techniques such as whole-genome sequencing were not routinely employed, limiting insights into transmission dynamics. Overall, our findings underscore that risk factors for candidemia are species-specific. This has direct clinical implications for diagnosis, empirical therapy, and infection control. In the era of emerging multidrug-resistant species such as *C. auris*, targeted surveillance, antifungal stewardship, and species-level diagnostics are critical for optimizing patient outcomes and curbing hospital transmission.

## 5. Conclusions

In this 10-year study at a general hospital in Kuwait, we observed a rising incidence of candidemia and a marked shift in the causative species from a predominance of *C. albicans* to NAC spp. and related species, notably *C. auris* and *C. parapsilosis*. ICU admission, advanced age, and the presence of multiple comorbidities emerged as significant risk factors for candidemia. Notably, *C. auris* isolates exhibited high levels of resistance to antifungal agents, raising concern about the limited treatment options and necessitating robust antifungal stewardship efforts. These findings highlight the importance of early species identification and awareness of local susceptibility patterns to guide timely and appropriate therapy. They also underscore the need for stringent infection control measures to prevent the spread of resistant strains, particularly *C. auris*.

## Figures and Tables

**Table 1:** Distribution of candidemia patients in Al-Amiri Hospital, Kuwait (2015–2024).

Year	Total No. of Patients	Gender		No. Candidemia of Patients of Different Age (Years)					Hospital Unit	
		Male	Female	≤1	1–19	20–49	50–64	≥65	ICU	Ward
2015–2016	33	12	21	-	-	6	8	19	12 (36%)	21 (64%)
2017–2018	47	22	25	-	-	4	8	35	19 (40%)	28 (60%)
2019–2020	64	37	27	-	-	14	20	30	25 (38%)	39 (62%)
2021–2022	86	42	44	-	2	14	19	51	40 (47%)	46 (53%)
2023–2024	93	48	45	-	-	14	24	55	36 (39%)	57 (61%)
Total	323	161 (49.8%)	162 (50.2%)	-	2	52	79	190	132 (41%)	191 (59%)

**Table 2:** Age and gender distribution of patients diagnosed with candidemia.

Age of Candidemia Patients (Years)	*Candida* spp. and Related Species Isolates Identified as												Total
	*C. albicans*, n = 101		*C. parapsilosis*, n = 52		*C. tropicalis*, n = 46		*C. auris*, n = 45		*N. glabratus*, n = 58		Others n = 21		
	M	F	M	F	M	F	M	F	M	F	M	F	
≤1	-	-	-	-	-	-	-	-	-	-	-	-	-
1–19	-	-	-	-	2	-	-	-	-	-	-	-	2
20–49	2	8	5	5	7	2	8	1	7	6	-	1	52
50–64	17	6	6	9	3	4	10	5	7	7	3	2	79
≥65	32	36	11	16	12	16	8	13	14	17	7	8	190
Total	51	50	22	30	24	22	26	19	28	30	10	11	323

**Table 3:** Spectrum of *Candida* spp. and related species isolated from candidemia patients in Al-Amiri Hospital, Kuwait (2015–2024).

Organism	2015–2016	2017–2018	2019–2020	2021–2022	2023–2024
*Candida albicans*	19	18	26	21	17
*Candidozyma auris*	0	1	7	15	22
*Nakaseomyces glabratus*	4	8	13	17	16
*Candida parapsilosis*	2	5	8	13	24
*Candida tropicalis*	6	11	8	11	10
Others *	2	4	2	9	4
Total	33	47	64	86	93

* *Candida ciferrii*, n = 1; *Candida dubliniensis*, n = 4; *Candididozyma haemuli* (formerly *Candida haemulonii*), n = 3; *Pichia kudriavzevii*, n = 5; *Yarrowia lipolytica* (formerly *Candida lipolytica*), n = 1; *Clavispora lusitaniae* (formerly *Candida lusitaniae*), n = 3; *Cyberlindnera jadinii* (formerly *Candida utilis*), n = 2; *Kodamaea ohmeri*, n = 2.

**Table 4:** Antifungal susceptibility profiles of *Candida* spp. and related species isolated from bloodstream.

Organism	Antifungal	Range	GM	MIC50	MIC90	No. Resistant Isolates (%)
*Candida albicans* (14)						
	Fluconazole	0.25–256	0.862	0.5	16	2 (14.3%)
	Posaconazole	0.008–0.03	0.011	0.008	0.015	0 (0.0%)
	Voriconazole	0.008–0.03	0.011	0.008	0.015	0 (0.0%)
	Amphotericin B	0.12–1	0.247	0.25	0.5	0 (0.0%)
	Anidulafungin	0.015–0.12	0.042	0.06	0.06	0 (0.0%)
	Caspofungin	0.06–0.25	0.121	0.12	0.25	0 (0.0%)
	Micafungin	0.008–8	0.067	0.03	1	0 (0.0%)
	5-Flucytosine	<0.06–0.25	0.06	0.06	0.06	0 (0.0%)
*Candidozyma auris* * (39)						
	Fluconazole	0.25–256	70.28	64	256	38 (97.4%)
	Posaconazole	0.008–1	0.048	0.06	0.12	N/A
	Voriconazole	0.06–8	0.401	0.5	1	5 (12.8%)
	Amphotericin B	0.25–2	1	1	2	1 (2.6%)
	Anidulafungin	0.06–2	0.162	0.12	0.12	3 (7.7%)
	Caspofungin	0.12–8	0.265	0.25	1	2 (5.1%)
	Micafungin	0.015–4	0.083	0.06	0.12	2 (5.1%)
	5-Flucytosine	<0.06–64	0.121	0.06	0.12	1 (2.6%)
*Nakaseomyces glabratus* (16)						
	Fluconazole	0.25–64	3.67	4	64	4 (25.0%)
	Posaconazole	0.25–8	1.382	1	8	N/A
	Voriconazole	0.12–4	0.499	0.25	4	0 (0.0%)
	Amphotericin B	0.25–0.5	0.439	0.5	0.5	0 (0.0%)
	Anidulafungin	0.03–0.12	0.063	0.06	0.12	0 (0.0%)
	Caspofungin	0.12–64	0.627	0.12	8	0 (0.0%)
	Micafungin	0.015–1	0.031	0.015	0.5	0 (0.0%)
	5-Flucytosine	<0.06	0.06	0.06	0.06	0 (0.0%)
*Candida parapsilosis* (12)						
	Fluconazole	0.12–128	0.791	0.5	2	1 (8.3%)
	Posaconazole	0.015–0.12	0.048	0.045	0.12	N/A
	Voriconazole	0.008–0.06	0.015	0.008	0.06	0 (0.0%)
	Amphotericin B	0.12–2	0.311	0.25	1	0 (0.0%)
	Anidulafungin	0.06–2	0.442	0.5	2	0 (0.0%)
	Caspofungin	0.12–2	0.498	0.5	2	0 (0.0%)
	Micafungin	0.008–2	0.332	0.5	1	0 (0.0%)
	5-Flucytosine	<0.06–0.5	0.091	0.06	0.5	0 (0.0%)
*Candida tropicalis* (16)						
	Fluconazole	0.5–4	1.587	1	4	0 (0.0%)
	Posaconazole	0.03–0.5	0.108	0.12	0.5	N/A
	Voriconazole	0.015–0.25	0.08	0.09	0.25	0 (0.0%)
	Amphotericin B	0.5	0.5	0.5	0.5	0 (0.0%)
	Anidulafungin	0.03–0.5	0.096	0.06	0.5	0 (0.0%)
	Caspofungin	0.06–0.25	0.174	0.25	0.25	0 (0.0%)
	Micafungin	0.03–0.5	0.068	0.03	0.5	0 (0.0%)
	5-Flucytosine	<0.06	0.06	0.06	0.06	0 (0.0%)

* *C. auris* breakpoints are tentative (per CDC/CLSI guidelines); (GM) geometric mean; (MIC50) MIC inhibiting 50% of organisms; (MIC90) MIC inhibiting 90% of organisms.

**Table 5:** Multivariate analysis of risk factors for *Candida* species-specific candidemia and associated mortality *.

Risk Factor	*C. albicans* OR (95% CI)	*p*-Value	*N. glabratus* OR (95% CI)	*p*-Value	*C. auris* OR (95% CI)	*p*-Value	*C. parapsilosis* OR (95% CI)	*p*-Value	Mortality OR (95% CI)	*p*-Value
**Gender, male**	1.04 (0.65–1.66)	0.905	0.95 (0.54–1.67)	0.886	1.45 (0.77–2.74)	0.265	1.05 (0.58–1.89)	0.870	0.60 (0.38–0.93)	0.025
**Age (per 10-year)**	75 (32–88)		69 (39–92)		58 (28–92)		68 (16–104)		1.17 (0.96–1.42)	0.033
**Cancer**	5.94 (2.86–12.90)	<0.001	3.32 (1.68–6.50)	<0.001	0.33 (0.10–0.87)	0.016	0.75 (0.40–1.41)	0.370	0.23 (0.12–0.41)	<0.001
**Chronic kidney disease**	3.97 (1.62–10.21)	0.001	0.88 (0.43–1.74)	0.749	0.09 (0.02–0.31)	<0.001	0.92 (0.48–1.78)	0.810	2.62 (1.62–4.27)	<0.001
**Cerebrovascular accident**	2.73 (0.68–11.61)	0.105	0.50 (0.12–1.50)	0.263	1.36 (0.51–3.28)	0.490	1.32 (0.52–3.36)	0.560	1.44 (0.77–2.74)	0.232
**Ischemic heart disease**	4.43 (1.76–11.85)	<0.001	0.95 (0.46–1.88)	1.000	0.77 (0.35–1.60)	0.501	1.14 (0.63–2.07)	0.670	3.03 (1.86–5.00)	<0.001
**Hypertension**	2.79 (1.33–5.91)	0.005	2.92 (1.57–5.54)	<0.001	0.31 (0.14–0.64)	<0.001	1.22 (0.68–2.19)	0.510	1.71 (1.07–2.75)	0.024
**Diabetes mellitus**	2.38 (1.17–4.82)	0.012	1.69 (0.92–3.15)	0.081	0.44 (0.22–0.88)	0.014	1.09 (0.60–1.99)	0.780	1.26 (0.77–2.05)	0.351
**Dyslipidemia**	2.79 (1.18–6.68)	0.010	2.06 (1.10–3.86)	0.017	0.29 (0.11–0.65)	<0.001	1.31 (0.73–2.36)	0.370	1.58 (0.99–2.53)	0.043
**Surgery**	1.56 (0.77–3.13)	0.224	6.08 (2.95–13.61)	<0.001	0.69 (0.35–1.38)	0.323	2.91 (1.63–5.19)	0.008	0.41 (0.24–0.68)	<0.001
**Mechanical ventilation**	3.36 (1.12–10.75)	0.016	0.29 (0.07–0.84)	0.014	0.68 (0.26–1.58)	0.448	1.18 (0.66–2.11)	0.580	1.91 (1.12–3.29)	0.015
**Total parenteral nutrition**	2.67 (0.75–9.90)	0.122	0.85 (0.30–2.07)	0.834	1.04 (0.39–2.45)	1.000	3.71 (1.93–7.14)	0.002	1.41 (0.78–2.55)	0.261
**Central venous catheter**	2.99 (0.96–9.77)	0.048	10.57 (5.34–21.38)	<0.001	0.75 (0.29–1.76)	0.564	4.18 (2.34–7.49)	<0.001	1.64 (0.95–2.84)	0.068
**Prolonged IV therapy (>7 d)**	–	–	–	–	–	–	2.43 (1.36–4.34)	0.015	–	–
**Urinary catheter**	3.35 (0.38–40.77)	0.179	0 (0–1.08)	0.050	0 (0–0.98)	0.034	1.55 (0.84–2.86)	0.160	1.76 (0.71–4.53)	0.213

* CVA, central venous access; IHD, ischemic heart disease; HTN, hypertension. *N. glabratus* was independently associated with cancer (OR: 3.32; *p* < 0.001), HTN (OR: 2.92; *p* <10 0.001), dyslipidemia (OR: 2.06; *p* = 0.017), surgery (OR: 6.08; *p* < 0.001), ventilator use (protective; OR: 0.29; *p* = 0.014), and central venous catheterization (OR: 10.57; *p* < 0.001).

## Data Availability

All relevant data are within the manuscript.

## Abbreviations

The following abbreviations are used in this manuscript:

MIC	Minimum inhibitory concentration
NAC	Non-*albicans* *Candida*
